# Primary Retroperitoneal Hydatid Cyst in a Pediatric Patient: A Report of a Rare Case

**DOI:** 10.7759/cureus.101351

**Published:** 2026-01-12

**Authors:** Hafid Talha

**Affiliations:** 1 Laboratory of Health Sciences, Faculty of Medicine and Pharmacy of Errachidia, Moulay Ismail University of Meknes, Errachidia, MAR; 2 Pediatric Surgery, Moulay Ali Cherif Regional Hospital Center, Errachidia, MAR

**Keywords:** abdominal hydatid cyst, cystic hydatidosis, echinococcus granulosus, hydatid cyst, retroperitoneal hydatid cyst

## Abstract

Hydatid disease is a parasitic infection caused by the larval stage of *Echinococcus granulosus*; it can involve any organ. Retroperitoneal localization is exceptionally rare and often presents with nonspecific symptoms, which may delay diagnosis and mimic other retroperitoneal cystic masses. Therefore, in endemic settings, hydatid disease should be considered in the differential diagnosis of any retroperitoneal mass. We report a 13-year-old boy who presented with an isolated, painless right flank mass. Non-contrast abdominal CT showed a well-defined, right subhepatic retroperitoneal cystic. The patient underwent complete surgical excision via an extraperitoneal lumbotomy.

This case highlights the importance of maintaining clinical suspicion for hydatid disease when evaluating retroperitoneal cystic masses in endemic areas and supports careful surgical management to prevent intraoperative dissemination.

## Introduction

Hydatid disease or cystic echinococcosis remains a common and well-recognized parasitic infection in endemic regions, including Morocco [1]. Caused by the larval stage of *Echinococcus granulosus*, it can involve any organ in the human body. Most cases predominantly affect the liver and lungs, and involvement of other sites is uncommon, which can further complicate clinical suspicion. Retroperitoneal involvement is exceptionally rare [2]. Because symptoms are often nonspecific, the diagnosis may be delayed, and the condition may mimic other retroperitoneal masses [1]. In endemic settings, a hydatid cyst should therefore be considered in the differential diagnosis of any retroperitoneal mass [3]. Complete surgical excision with careful avoidance of cyst rupture remains the cornerstone of treatment to reduce recurrence and complications [4]. Given the rarity of retroperitoneal hydatid cysts, documenting this case helps expand the limited clinical literature on this location and reinforces diagnostic vigilance for atypical presentations.

## Case presentation

We present here the case of a 13-year-old boy who presented (11/2024) with a right flank mass, without associated symptoms. On physical examination, a painless, palpable mass was noted in the right flank, measuring approximately 6 × 5 cm. The mass was mobile relative to the superficial plane but appeared fixed to the deep plane. The remainder of the clinical examination was unremarkable. Laboratory testing showed a normal white blood cell count, normal C-reactive protein level, and normal hemoglobin level. Renal function parameters (urea and creatinine) were normal, and hemostasis was normal with a normal prothrombin time (Table 1).

**Table 1:** Summary of relevant admission laboratory results

Parameter	Value	Normal Range
White blood cell count	7.0 × 10^9^/L	4.5-13.5 × 10^9^/L
C-reactive protein (CRP)	2 mg/L	<5 mg/L
Hemoglobin	14.0 g/dL	12.0-16.0 g/dL
Urea	0.30 g/L	0.15-0.45 g/L
Creatinine	8 mg/L	5-12 mg/L
Prothrombin time (PT)	90%	70-100%

**Figure 1:**
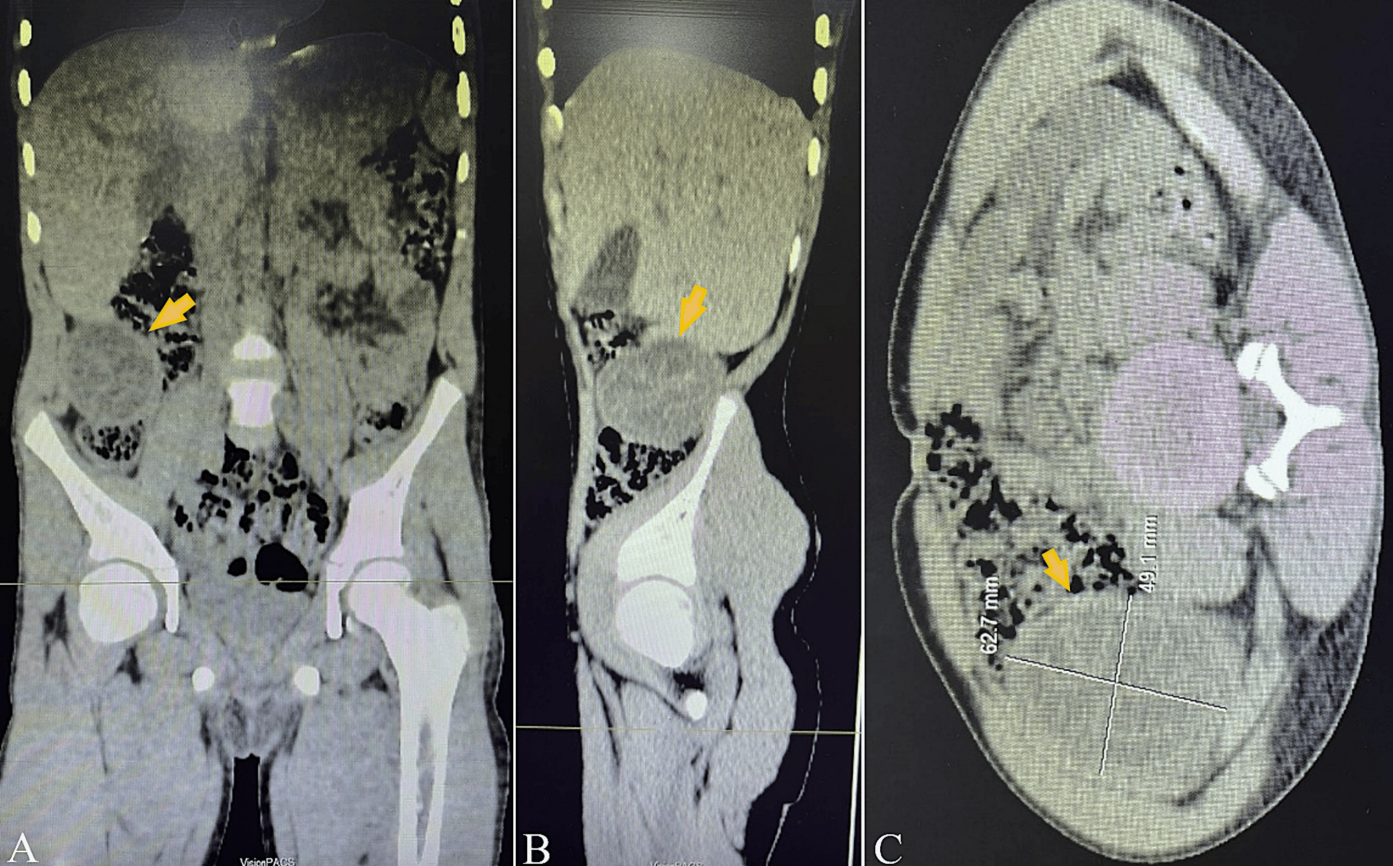
Non-contrast abdominal CT demonstrating a well-circumscribed right retroperitoneal cystic mass measuring 62.7 mm in maximal diameter on coronal (A), sagittal (B), and axial (C) images (arrows).

Imaging findings of a non-contrast abdominal computed tomography (CT) scan demonstrated a well-circumscribed, round cystic lesion in the right retroperitoneal subhepatic region, with regular margins. An internal detached membrane was visible within the cyst. The lesion measured 62.7 × 49.1 mm (Figure 1).

**Figure 2:**
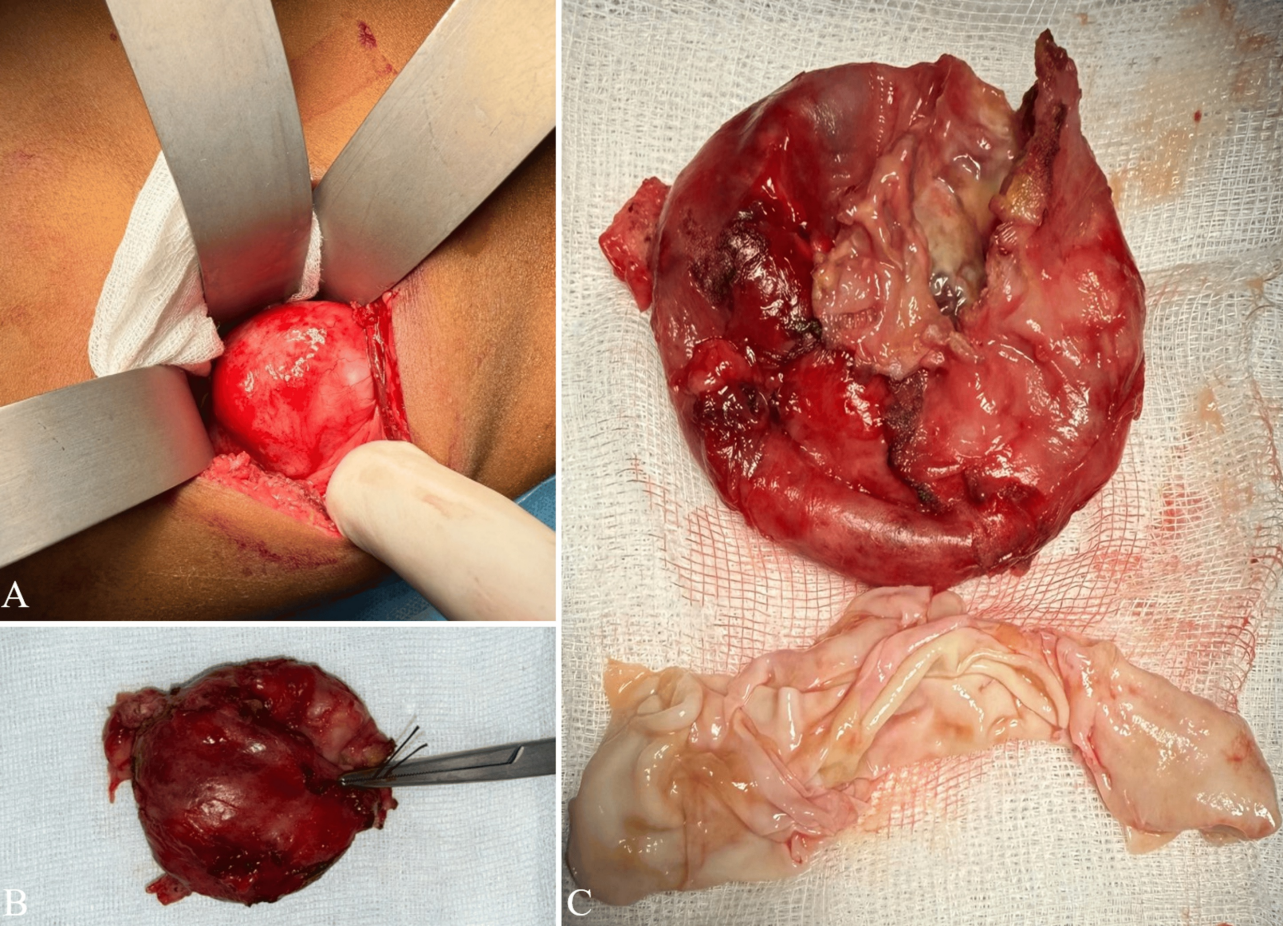
Intraoperative photographs demonstrating the cystic mass after extraperitoneal lumbotomy (A) and the gross appearance of the resected specimen before opening (B) and after cyst incision (C), showing the fibrous wall at the top and hydatid membranes at the bottom.

The patient underwent surgery under general anesthesia in the left lateral decubitus position. An extraperitoneal lumbar approach (lumbotomy) was performed. After isolating the mass, the operative field was protected using a scolicidal agent (30% hypertonic saline). The mass was carefully dissected with release of parietal adhesions, followed by complete excision (Figure 2). Hemostasis was secured, and the abdominal wall was closed in layers. The patient didn’t show any postoperative complications.

**Figure 3:**
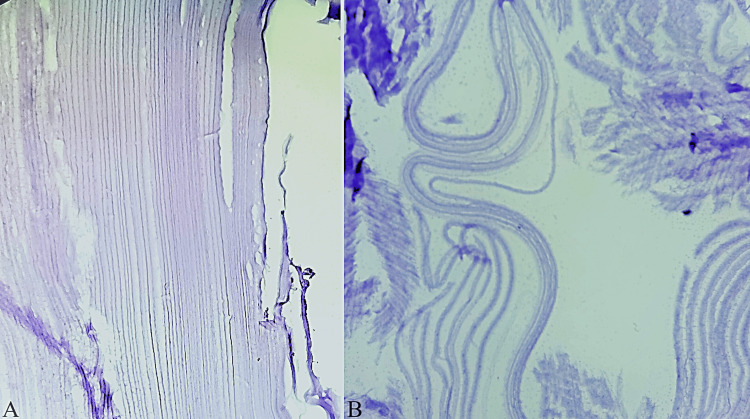
Microphotographs showing an acellular laminated membrane with characteristic striations, consistent with a hydatid cyst; no scolices were identified (A and B).

Histopathologic examination showed an acellular laminated membrane with characteristic striations, consistent with a hydatid cyst. No scolices were identified (Figure 3).

The patient was discharged in good condition. At one-year follow-up, he remained asymptomatic, with no clinical or radiologic evidence of recurrence.

## Discussion

Hydatid disease is a parasitic infection caused by the larval stage of *E. granulosus* [1]. The liver and lungs are the most frequently involved organs, reflecting the typical hematogenous dissemination after intestinal absorption; however, cysts may rarely develop in atypical sites, sometimes without detectable hepatic or pulmonary involvement [1,4].

The disease remains endemic in several regions, including Southeast Asia, the Mediterranean basin, and the Middle East [5], where it represents an important public health problem. Humans are accidental intermediate hosts and become infected by ingesting parasite eggs through contaminated food or water [6].

Because retroperitoneal involvement is uncommon, particularly in the absence of concomitant liver or lung disease, it is less likely to be suspected when evaluating a retroperitoneal mass [1]. Primary retroperitoneal hydatid cysts are exceptionally rare, reported to account for <0.5% of all cases [7]. Clinical manifestations are usually nonspecific and depend largely on cyst size and location. Patients may present with abdominal or lumbar pain, a slowly enlarging mass, or symptoms related to compression of adjacent structures [4]. Lesions may remain silent for a prolonged period and are sometimes discovered only after complications such as rupture or secondary bacterial infection, which can be life-threatening in the setting of anaphylaxis or sepsis [8].

The differential diagnosis of a retroperitoneal cystic mass is broad and includes retroperitoneal neoplasms such as teratoma, lymphoma, and sarcoma, as well as other benign or inflammatory conditions. Establishing a preoperative diagnosis is therefore essential to guide appropriate management and minimize the risk of inadvertent spillage or incomplete treatment [1]. Cross-sectional imaging, particularly CT, is central to improving pretherapeutic diagnostic accuracy. Serologic testing may be supportive, but a negative result does not exclude hydatid disease [9]. In our patient, the imaging appearance combined with the endemic context strongly suggested the diagnosis preoperatively.

Percutaneous aspiration or puncture is generally discouraged in retroperitoneal hydatid cysts because of the risk of dissemination and, in rare cases, anaphylactic reaction [1]. Surgery remains the cornerstone of treatment and should be performed with strict precautions to prevent intraoperative spillage, including isolation of the cyst from surrounding tissues using gauze packs soaked in a scolicidal solution [10]. Perioperative antiparasitic therapy with albendazole may be used to reduce the risk of recurrence, and postoperative medical therapy is often recommended as an adjunct in selected cases [1,11].

This case underscores the importance of including hydatid disease in the differential diagnosis of retroperitoneal cystic lesions, particularly in endemic areas, even when there is no evidence of hepatic or pulmonary involvement.

## Conclusions

Primary retroperitoneal hydatid disease is an exceptionally rare presentation of cystic echinococcosis, particularly in children and in the absence of hepatic or pulmonary involvement. In endemic regions such as Morocco, a hydatid cyst should be included in the differential diagnosis of any retroperitoneal cystic mass, even when clinical manifestations are minimal or nonspecific. Cross-sectional imaging, especially CT, can strongly suggest the diagnosis, but histopathology remains confirmatory. Complete surgical excision with strict measures to prevent intraoperative spillage is the cornerstone of treatment, and adjunctive perioperative albendazole may help reduce recurrence risk. This case highlights that retroperitoneal cystic masses should prompt consideration of hydatid disease in endemic regions, even without liver or lung involvement.
